# Inflammasome Activation by Bacterial Outer Membrane Vesicles Requires Guanylate Binding Proteins

**DOI:** 10.1128/mBio.01188-17

**Published:** 2017-10-03

**Authors:** Ryan Finethy, Sarah Luoma, Nichole Orench-Rivera, Eric M. Feeley, Arun K. Haldar, Masahiro Yamamoto, Thirumala-Devi Kanneganti, Meta J. Kuehn, Jörn Coers

**Affiliations:** a Department of Molecular Genetics and Microbiology, Duke University Medical Center, Durham, North Carolina, USA; b Department of Biochemistry, Duke University Medical Center, Durham, North Carolina, USA; c Department of Immunoparasitology, Research Institute for Microbial Diseases, Osaka University, Osaka, Japan; d Department of Immunology, St. Jude Children’s Research Hospital, Memphis, Tennessee, USA; e Department of Immunology, Duke University Medical Center, Durham, North Carolina, USA; Emory Vaccine Center; Harvard School of Public Health

**Keywords:** GBP2, GBP5, LPS, OMVs, caspase-11, guanylate binding proteins, inflammasome, interferons, lipopolysaccharide, outer membrane vesicles, sepsis

## Abstract

The Gram-negative bacterial cell wall component lipopolysaccharide (LPS) is recognized by the noncanonical inflammasome protein caspase-11 in the cytosol of infected host cells and thereby prompts an inflammatory immune response linked to sepsis. Host guanylate binding proteins (GBPs) promote infection-induced caspase-11 activation in tissue culture models, and yet their *in vivo* role in LPS-mediated sepsis has remained unexplored. LPS can be released from lysed bacteria as “free” LPS aggregates or actively secreted by live bacteria as a component of outer membrane vesicles (OMVs). Here, we report that GBPs control inflammation and sepsis in mice injected with either free LPS or purified OMVs derived from Gram-negative *Escherichia coli*. In agreement with our observations from *in vivo* experiments, we demonstrate that macrophages lacking GBP2 expression fail to induce pyroptotic cell death and proinflammatory interleukin-1β (IL-1β) and IL-18 secretion when exposed to OMVs. We propose that in order to activate caspase-11 *in vivo*, GBPs control the processing of bacterium-derived OMVs by macrophages as well as the processing of circulating free LPS by as-yet-undetermined cell types.

## IMPORTANCE

The bacterial cell wall component LPS is a strong inducer of inflammation and is responsible for much of the toxicity of Gram-negative bacteria. Bacteria shed some of their cell wall and its associated LPS in the form of outer membrane vesicles (OMVs). Recent work demonstrated that secreted OMVs deliver LPS into the host cell cytosol by an unknown mechanism, resulting in the activation of the proinflammatory LPS sensor caspase-11. Here, we show that activation of cytosolic caspase-11 by OMVs requires additional host factors, the so-called guanylate binding proteins (GBPs). The discovery of GBPs as regulators of OMV-mediated inflammation paves the way toward a mechanistic understanding of the host response toward bacterial OMVs and may lead to effective strategies to ameliorate inflammation induced by bacterial infections.

## INTRODUCTION

Lipopolysaccharide (LPS) is an essential building block of all Gram-negative bacteria and a potent inducer of sepsis (1). Extracellular and endocytosed LPS is recognized by the transmembrane protein Toll-like receptor 4 (TLR4) and prompts discrete signaling events originating from the plasma membrane and from endosomes (2, 3). LPS within the host cell cytosol is detected by caspase-11, a second LPS receptor, which initiates the execution of proinflammatory cell death (pyroptosis) and the processing and secretion of proinflammatory cytokines interleukin-1β (IL-1β) and IL-18 by caspase-1 (4, 5). Therefore, compartmentalization of LPS receptors allows host cells to respond differentially to the presence of LPS at three distinct subcellular locales.

Long thought to be the sole LPS sensor in mammals, TLR4 has been studied in great detail, leading to the discovery and characterization of several auxiliary proteins required for the activation of TLR4 signaling (2). The discovery of the noncanonical inflammasome protein caspase-11 in mice and CASP-4/-5 in humans as cytosolic sensors for LPS is more recent (6), and accordingly, little is known about the existence of additional host factors controlling its activity. One notable exception is the regulation of caspase-11-dependent host responses by guanylate binding proteins (GBPs). GBPs constitute a family of proteins induced by interferon gamma (IFN-γ) as well as type I IFNs. GBPs exhibit potent antimicrobial activities both in cell culture models and *in vivo* (7–9). GBPs were additionally shown to promote caspase-11 activation in macrophages infected with *Salmonella*, *Legionella*, or *Chlamydia* (10–12), all Gram-negative bacteria that occupy pathogen-containing vacuoles (PVs) inside infected host cells (13). Mechanistic *in vitro* studies led to an attractive model, according to which GBPs lyse PVs and thereby release bacteria and their associated LPS into the host cell cytosol for recognition by caspase-11 (10). This model was based on results obtained from *Salmonella* infection studies in cultured macrophages (10) but is contradicted by our own studies using *Legionella* and *Chlamydia* infection models (11, 12). Thus, while GBPs have been identified as key regulators of caspase-11 function, the mechanism by which they do so remains controversial.

Because caspase-11 is a cytosolic LPS receptor (6, 14), its activation depends on the delivery of LPS across host cell membranes into the host cell cytosol. Injection of purified LPS into mice activates caspase-11 *in vivo* (4, 5), thus arguing that mechanisms exist by which circulating LPS can traverse eukaryotic membranes and enter the host cell cytosol. However, the nature of these mechanisms is unknown, and their existence may be restricted to specific cell types. Indeed, cultured macrophages lack the ability to import extracellular LPS into their host cell cytosol and therefore do not induce caspase-11 activation when purified “free” LPS is added extracellularly (4, 5). However, in contrast to free LPS, adding bacterial outer membrane vesicles (OMVs) comprised of LPS and other cell wall components to culture medium is sufficient to activate caspase-11 in macrophages, which are able to extract and import LPS from ingested OMVs into their cytosol by a poorly characterized mechanism (15). Because bacteria can produce immunomodulatory OMVs (16, 17), we hypothesized that GBP-dependent caspase-11 activation during macrophage infections was mediated by OMVs. In support of this hypothesis, we observe that GBPs are essential for OMV-induced pyroptosis and IL-1β/IL-18 secretion. Additionally, we find that GBP-deficient mice injected with OMVs or purified LPS display lower IL-1β/IL-18 serum levels and lower mortality rates than wild-type mice. Our studies thus reveal GBPs as critical regulators of inflammation induced by circulating free LPS or OMVs *in vivo*.

## RESULTS

### Avirulent *Escherichia coli* activates pyroptosis and interleukin secretion in a GBP-dependent manner.

**FIG 1:**
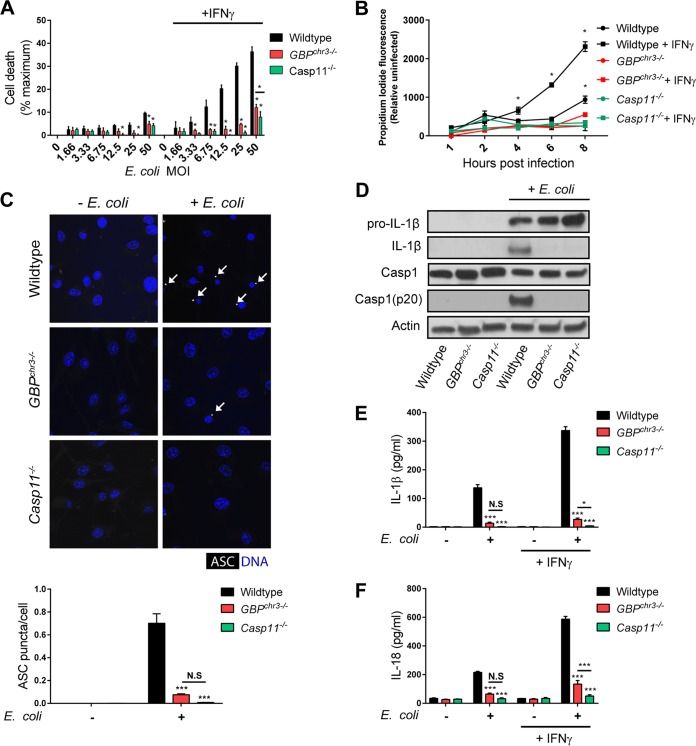
GBPs control noncanonical and canonical inflammasome activation in macrophages exposed to *E. coli*. Wild-type, *GBP*^chr3−/−^, and *Casp11*^−/−^ BMDMs were primed with IFN-γ overnight or left unprimed and then exposed to *E. coli* strain K-12 at the indicated MOI or left uninfected. (A) LDH release was measured at 8 hpi. (B) IFN-γ-primed and unprimed BMDMs were infected with *E. coli* at an MOI of 25, and propidium iodide fluorescence resulting from nuclear incorporation in dead cells was measured at the indicated times following infection. (C) IFN-γ-primed BMDMs were either left untreated or infected with *E. coli* at an MOI of 25 for 8 h and subsequently stained with anti-ASC antibody and Hoechst stain (for DNA/nuclei). White arrows indicate ASC specks. The number of ASC puncta per cell was quantified. (D) IFN-γ-primed BMDMs were infected with *E. coli* at an MOI of 25, and at 8 hpi cell lysates and supernatants were collected. Protein levels in total cell lysates (caspase-1, pro-IL-1β, and actin) or supernatant (IL-1β and caspase-1 p20) were visualized via immunoblotting. (E and F) IL-1β (E) and IL-18 (F) concentrations were measured by ELISA in culture supernatants collected from BMDMs exposed to *E. coli* for 8 h (MOI of 25). Data shown are means ± SEM from 3 independent experiments. The statistical significance shown is relative to the wild type (A, C, E, and F) or relative to *GBP*^chr3−/−^ BMDMs (B) of the same experimental groups, unless indicated otherwise. Significance is indicated as follows: ***, *P* < 0.001; **, *P* < 0.01; *, *P* < 0.05; N.S, not statistically significant (two-way ANOVA with Tukey’s multiple-comparison test).

Caspase-11 activation in response to infections with Gram-negative bacterial pathogens is diminished in macrophages derived from *GBP*^chr3−/−^ mice, in which the *Gbp* gene cluster on chromosome 3 is deleted (10–12). To determine whether GBPs were also required for caspase-11 activation by nonpathogenic Gram-negative bacteria, we monitored caspase-11-dependent cellular responses in *GBP*^chr3−/−^ bone marrow-derived macrophages (BMDMs) exposed to the nonpathogenic *Escherichia coli* strain K-12. Because bacterial burden could potentially impact caspase-11 activation, we first monitored *E. coli* K-12 survival inside wild-type and *GBP*^chr3−/−^ BMDMs. We found that the absence of GBPs had no impact on the number of retrievable CFU in IFN-γ-primed BMDMs at 4 h postinfection (hpi) (see Fig. S1 in the supplemental material), indicating that changes in bacterial burden are unlikely to account for any phenotypes associated with the *GBP*^chr3−/−^ genotype at this or later times postinfection. Next, we investigated whether activation of the noncanonical inflammasome was altered in *GBP*^chr3−/−^ BMDMs. Whereas unprimed BMDMs exposed to *E. coli* K-12 failed to undergo marked cell death at 8 hpi across a broad range of multiplicities of infection (MOIs), we found that IFN-γ-primed wild-type but not *GBP*^chr3−/−^ or *Casp11*^*−/−*^ BMDMs rapidly succumbed to *E. coli*-triggered cell death, as measured by lactose dehydrogenase (LDH) release (Fig. 1A) or host cell nuclear incorporation of propidium iodide (Fig. 1B). These observations demonstrate that GBPs are required for the induction of caspase-11-dependent pyroptosis by nonpathogenic *E. coli*.

10.1128/mBio.01188-17.1 FIG S1  Viability of phagocytosed *E. coli* is independent of *Gbp* gene cluster on chromosome 3. IFN-γ-primed wild-type, *GBP*^chr3−/−^, and *Casp11*^−/−^ BMDMs were infected with *E. coli* at an MOI of 25, and recoverable CFU were determined at 1 and 4 hpi. Mean ± standard deviation is shown. Each symbol represents an individual replicate. Significance was defined as follows: ***, *P* < 0.001; **, *P* < 0.01; *, *P* < 0.05; N.S, not statistically significant (two-way ANOVA with Sidak’s multiple-comparison test). Download FIG S1, TIF file, 0.2 MB. Copyright © 2017 Finethy et al. 2017 Finethy et al. This content is distributed under the terms of the Creative Commons Attribution 4.0 International license.

In addition to the induction of pyroptosis, caspase-11 activation promotes the formation of the canonical NLR family pyrin domain-containing 3 (NLRP3) inflammasome (18). Because GBPs were required for caspase-11-dependent pyroptosis (Fig. 1A and B), we tested their role in the activation of the canonical inflammasome in *E. coli*-infected BMDMs. A hallmark of canonical inflammasome assembly is the formation of intracellular foci comprised of the adapter protein apoptosis-associated speck-like protein containing a CARD (ASC). We noticed that the number of cells with ASC specks was significantly reduced in *GBP*^chr3−/−^ BMDMs compared to wild-type BMDMs (Fig. 1C). Similarly, processed caspase-1 and IL-1β were detectable by Western blotting in cell supernatants of *E. coli*-infected wild-type BMDMs but undetectable in cell supernatants of *Casp11*^*−/−*^ and *GBP*^chr3−/−^ BMDMs (Fig. 1D). Third, both IL-1β and IL-18 secretion in response to *E. coli* infections was dramatically diminished in *GBP*^chr3−/−^ BMDMs (Fig. 1E and F). Because *GBP*^chr3−/−^ BMDMs by and large phenocopied *Casp11*^*−/−*^ BMDMs in all of these functional assays (Fig. 1), we conclude that GBPs are critical for the induction of the pyroptotic death pathway and the activation of the NLRP3 inflammasome by caspase-11 in *E. coli-*infected BMDMs.

### Induction of pyroptosis and interleukin secretion by OMVs requires GBPs.

**FIG 2:**
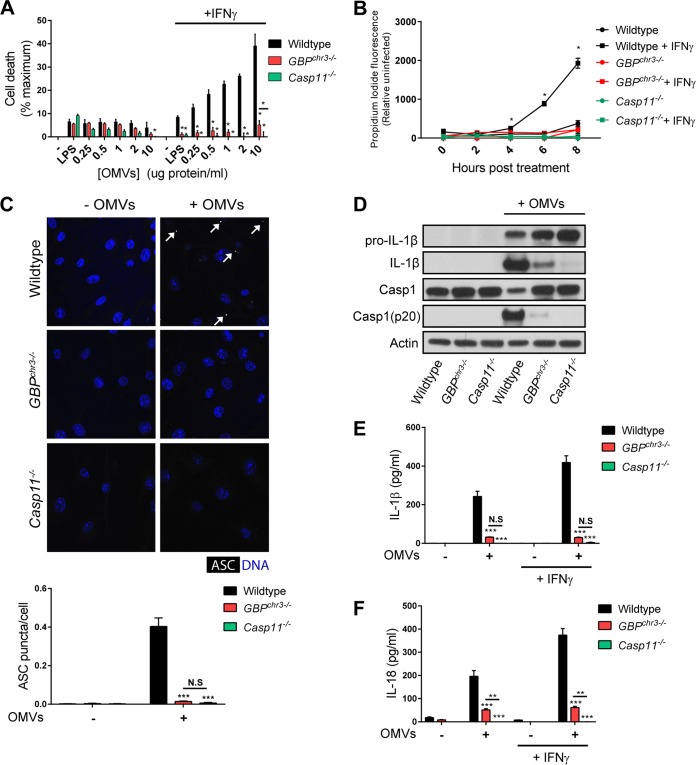
GBPs control noncanonical and canonical inflammasome activation in macrophages exposed to OMVs. Wild-type, *GBP*^chr3−/−^, and *Casp11*^−/−^ BMDMs were primed with 100 U/ml IFN-γ overnight or left unprimed and then treated with 1 µg/ml LPS or *E. coli*-derived OMVs at the indicated protein concentrations. (A) LDH release was measured at 8 h posttreatment. (B) IFN-γ-primed and unprimed BMDMs were treated with 2 µg/ml OMVs, and propidium iodide fluorescence was measured at the indicated times following treatment. (C) IFN-γ-primed BMDMs were either treated with OMVs (2 µg/ml) for 8 h or left untreated and were subsequently stained with anti-ASC antibody and Hoechst stain (nuclei), and the number of ASC puncta (white arrows) per cell was quantified. (D) IFN-γ-primed BMDMs were treated with OMVs (2 µg/ml), and at 8 h posttreatment, cell lysates and supernatants were collected. Protein levels in total cell lysates (caspase-1, pro-IL-1β, and actin) or supernatant (IL-1β and caspase-1 p20) were visualized via immunoblotting. (E and F) IL-1β (E) and IL-18 (F) concentrations were measured by ELISA in culture supernatants collected from BMDMs treated with OMVs (2 µg/ml) for 8 h. Data shown are means ± SEM from 3 independent experiments. The statistical significance shown is relative to the wild type (A, C, E, and F) or relative to *GBP*^chr3−/−^ BMDMs (B) of the same experimental groups, unless indicated otherwise. Significance was defined as follows: ***, *P* < 0.001; **, *P* < 0.01; *, *P* < 0.05; N.S, not statistically significant (two-way ANOVA with Tukey’s multiple-comparison test).

Previous studies proposed that GBPs extract LPS from intracellular bacterial pathogens through PV lysis and bacteriolysis, processes that require direct binding of GBPs to PVs or bacteria, respectively (10, 19–21). In agreement with our recent finding that GBPs specifically detect phagosomes occupied by virulent bacteria but fail to associate with phagosomes containing avirulent bacteria (22), we observed that the association of GBP2 with phagosomes containing *E. coli* K-12 was infrequent (Fig. S2). This observation suggested an alternative mechanism by which GBPs promote caspase-11 activation in macrophages exposed to *E. coli*. Recently, it was reported that OMVs are the predominant inducer of caspase-11 activation through *E. coli* infections (15). Because GBPs are essential for *E. coli*-induced caspase-11 activation (Fig. 1), we asked whether GBPs were also required for inflammasome activation in response to purified OMVs added to macrophages extracellularly. As shown previously (4, 5), the addition of extracellular LPS, even at high concentrations (1 µg/ml), had minimal impact on cell viability in both unprimed and IFN-γ-primed BMDMs (Fig. 2A). In contrast to LPS, OMVs added to the medium of IFN-γ-primed BMDMs induced rapid, caspase-11-dependent cell death in a concentration-dependent manner (Fig. 2A and B). Similarly to *E. coli-*induced pyroptosis (Fig. 1), this OMV-induced cell death required the expression of GBPs (Fig. 2A and B). ASC speck formation (Fig. 2C), proteolytic processing of pro-caspase-1 and pro-IL-1β (Fig. 2D), and IL-1β and IL-18 secretion (Fig. 2E and F) triggered by the addition of OMVs were also dependent on GBPs. Together, these data show that pronounced activation of noncanonical and canonical inflammasome responses by extracellular OMVs requires GBPs.

10.1128/mBio.01188-17.2 FIG S2  GBP2 infrequently associates with phagocytosed *E. coli*. IFN-γ-primed BMDMs were infected with green fluorescent protein (GFP)-*Legionella pneumophila* or GFP-*E*. *coli* at an MOI of 1. Two hours postinfection, cells were stained with Hoechst stain for DNA (blue), and anti-GBP2 antibodies (red). Magnification, ×63. Frequencies at which GBP2 colocalizes with either *Legionella*- or *E. coli-*containing phagosomes were quantified from 3 independent experiments (>200 bacteria/replicate). Mean ± SEM is shown. Significance was defined as follows: *, *P* < 0.05 (unpaired *t* test). Download FIG S2, JPG file, 1.3 MB. Copyright © 2017 Finethy et al. 2017 Finethy et al. This content is distributed under the terms of the Creative Commons Attribution 4.0 International license.

### GBP2 but not GBP5 controls caspase-11-dependent pyroptosis and interleukin secretion in cultured macrophages.

**FIG 3:**
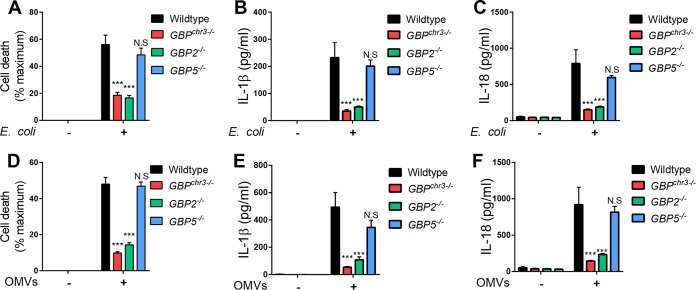
GBP2 promotes OMV-induced pyroptosis and IL-1β/IL-18 secretion in macrophages. (A to C) Wild-type, *GBP*^chr3−/−^, *GBP2*^−/−^, and *GBP5*^−/−^ BMDMs were primed with IFN-γ and infected with *E. coli* at an MOI of 25. At 8 hpi, LDH release (A) and IL-1β (B) and IL-18 (C) secretion were measured. IFN-γ-primed wild-type, *GBP*^chr3−/−^, *GBP2*^−/−^, and *GBP5*^−/−^ BMDMs were treated with *E. coli*-derived OMVs for 8 h. (D to F) Subsequently, LDH release (D) and IL-1β (E) and IL-18 (F) secretion were measured. Data shown are means ± SEM from 3 independent experiments (A and D) or 6 independent experiments (B, C, E, and F). The statistical significance shown is relative to the wild type. Significance was defined as follows: ***, *P* < 0.001; **, *P* < 0.01; *, *P* < 0.05; N.S, not statistically significant (two-way ANOVA with Tukey’s multiple-comparison test).

The chromosomal deletion in *GBP*^chr3−/−^ mice eliminates 5 *Gbp* genes, namely, *Gbp1*, *Gbp2*, *Gbp3*, *Gbp5*, and *Gbp7* (23). Previous studies demonstrated that expression of GBP2 promotes the induction of caspase-11-dependent cell death in macrophages infected with Gram-negative bacterial pathogens such as *Salmonella* and *Legionella* (10, 12). In support of a possible role for GBP2 in OMV processing, we detected partial colocalization between GBP2 and LPS in OMV-treated BMDMs (Fig. S3). We therefore tested whether GBP2 was required for caspase-11 activation by nonpathogenic *E. coli* and OMVs. We found that *GBP2*^−/−^ BMDMs mimicked *GBP*^chr3−/−^ BMDMs in their unresponsiveness to *E. coli* infections, as measured by LDH release as a marker of cell death (Fig. 3A) and secretion of IL-1β (Fig. 3B) and IL-18 (Fig. 3C). Similarly, GBP2 was essential for the robust induction of cell death (Fig. 3D) as well as IL-1β (Fig. 3E) and IL-18 (Fig. 3F) secretion by OMV treatment. Together, these data demonstrate that GBP2 is critical for OMV-mediated and caspase-11-dependent pyroptosis and canonical inflammasome activation.

10.1128/mBio.01188-17.3 FIG S3  GBP2 and LPS colocalize in OMV-treated BMDMs. IFN-γ-primed BMDMs were treated with OMVs (2 µg/ml) for 2 h and subsequently fixed and stained for LPS (green), GBP2 (red), and DNA (Hoechst stain, blue). Representative insets and line trace analysis are shown. White arrows indicate positions of corresponding fluorescence line tracing profiles. Magnification, ×63. Download FIG S3, JPG file, 1.7 MB. Copyright © 2017 Finethy et al. 2017 Finethy et al. This content is distributed under the terms of the Creative Commons Attribution 4.0 International license.

The specific activities of individual GBPs are poorly characterized, but a number of recent studies indicated that individual GBPs fulfill specialized, nonredundant functions in cell-autonomous immunity (24–29). One study proposed that GBP5 specifically assists assembly of the NLRP3 inflammasome and enhances responsiveness to a subset of NLRP3 priming agents (27). Based on these previous findings, we asked whether GBP5 modulated inflammasome activation in response to *E. coli* infections or OMV treatment in macrophages. We found that GBP5 was dispensable for *E. coli*- and OMV-induced pyroptosis as well as IL-1β and IL-18 secretion in BMDMs (Fig. 3). These data indicate that GBP5 is nonessential for canonical inflammasome responses that occur downstream from caspase-11 activation in cultured macrophages.

### GBPs control OMV- and LPS-induced inflammation *in vivo.*

**FIG 4:**
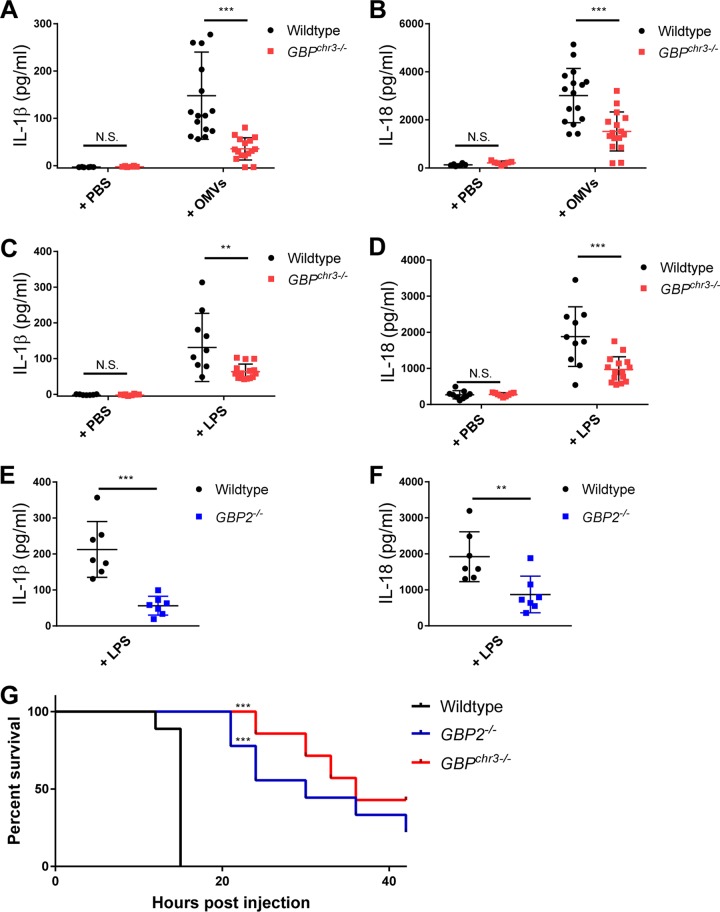
GBP-deficient mice display reduced IL-1β/IL-18 serum levels and increased survival rates during endotoxemia. (A and B) Wild-type and *GBP*^chr3−/−^ mice were injected i.p. with poly(I⋅C) at a dose of 2 mg/kg body weight and then 6 h later injected i.p. with 4 µg purified OMVs per mouse. Serum was obtained 6 h after OMV injection from OMV-injected wild-type (*n* = 16) or *GBP*^chr3−/−^ (*n* = 16) mice or from wild-type (*n* = 6) or *GBP*^chr3−/−^ (*n* = 7) mice injected with an equal volume of PBS, and IL-1β (A) and IL-18 (B) serum concentrations were measured by ELISA. (C and D) Wild-type (*n* = 10) and *GBP*^chr3−/−^ (*n* = 15) mice were injected with LPS (8 mg/kg body weight) or PBS alone (*n* = 9 for both wild-type and *GBP*^chr3−/−^ mice), and serum IL-1β (C) and IL-18 (D) were measured by ELISA 4 h postinjection. (E and F) Wild-type (*n* = 7) and *GBP2*^−/−^ (*n* = 7) mice were injected with LPS (8 mg/kg body weight), and serum IL-1β (E) and IL-18 (F) levels were measured by ELISA 4 h postinjection. (G) Wild-type (*n* = 9), *GBP*^chr3−/−^ (*n* = 7), and *GBP2*^−/−^ (*n* = 9) mice were i.p. injected with 2 mg/kg body weight of poly(I⋅C) and then 6 h later i.p. injected with LPS (20 mg/kg body weight). Morbidity and mortality were observed for 42 h at 3-h intervals. For panels A to F, mean ± standard deviation is shown. Each symbol represents an individual mouse. Significance was defined as follows: ***, *P* < 0.001; **, *P* < 0.01; *, *P* < 0.05; N.S, not statistically significant. Significance was measured by two-way ANOVA with Sidak’s multiple-comparison test (A to D), unpaired *t* test (E and F), or log rank test (G).

To assess the role of GBPs in caspase-11-dependent inflammation, we first injected mice with the TLR3 agonist poly(I⋅C), which prompts elevated expression of GBPs *in vivo* (Fig. S4) and skews LPS-triggered inflammation toward caspase-11- rather than TLR4-dominated responses (4, 5). According to previously established protocols (15), we first administered poly(I⋅C) for 6 h and then injected mice with OMVs and monitored IL-1β and IL-18 serum levels in wild-type and *GBP*^chr3−/−^ mice at 6 h post-OMV injection. We found that both IL-1β and IL-18 serum levels were significantly reduced in *GBP*^chr3−/−^ mice, indicating that GBPs promote OMV-induced inflammasome activation *in vivo* (Fig. 4A and B). We then assessed whether GBPs are also required for the activation of caspase-11 in response to *in vivo* administration of purified LPS. We found that *GBP*^chr3−/−^ (Fig. 4C and D) as well as *GBP2*^−/−^ (Fig. 4E and F) mice displayed lower IL-1β and IL-18 serum levels at 4 h postinjection. To monitor the role of GBPs in caspase-11-biased sepsis, poly(I⋅C)-treated mice received an injection of purified LPS at a concentration of 20 mg/kg body weight. We observed higher survival rates among *GBP*^chr3−/−^ and *GBP2*^−/−^ mice than among wild-type mice (Fig. 4G and S5). Together, these data demonstrate that GBPs are *in vivo* regulators of OMV- and LPS-induced inflammation and sepsis.

10.1128/mBio.01188-17.4 FIG S4  Systemic treatment with poly(I⋅C) induces GBP expression *in vivo*. Wild-type C57/BL6J mice were i.p. injected with poly(I⋅C) at a dose of 2 mg/kg body weight (*n* = 5) or PBS control (*n* = 5), and expression of GBP2 in the spleen was measured by qPCR and shown relative to PBS control group. Means ± SEM are shown. Significance was defined as follows: **, *P* < 0.01 (unpaired *t* test). Download FIG S4, TIF file, 0.1 MB. Copyright © 2017 Finethy et al. 2017 Finethy et al. This content is distributed under the terms of the Creative Commons Attribution 4.0 International license.

10.1128/mBio.01188-17.5 FIG S5  *GBP*^chr3−/−^ mice demonstrated increased survival under LPS-induced septic shock conditions. Wild-type (*n* = 10) and *GBP*^chr3−/−^ (*n* = 10) mice were i.p. injected with 2 mg/kg body weight of poly(I⋅C) and then 6 h later i.p. injected with LPS (20 mg/kg body weight). Morbidity and mortality were observed for 42 h at 3-h intervals. Significance was defined as follows: ***, *P* < 0.001 (log rank test). Download FIG S5, TIF file, 0.1 MB. Copyright © 2017 Finethy et al. 2017 Finethy et al. This content is distributed under the terms of the Creative Commons Attribution 4.0 International license.

## DISCUSSION

Caspase-11 directly detects LPS within the host cell cytosol (6). To explain how LPS gains access to the host cell cytosol, four distinct LPS delivery pathways were proposed: (i) some intracellular Gram-negative bacteria escape vacuoles to enter the host cell cytosol, where they release LPS (30); (ii) host GBPs execute membranolytic activities to extrude intracellular Gram-negative bacteria from PVs and extract LPS through bacteriolysis (10, 19–21); (iii) endocytosed bacterial OMVs release LPS into the host cell cytosol potentially through fusion with or transport across endosomal membranes (15, 31); and (iv) circulating free LPS (in the form of aggregates or bound to LPS-binding proteins) is consumed *in vivo* by an undefined cell population able to present LPS for caspase-11-mediated recognition (4, 5). Here, we present evidence that GBPs play previously unknown roles in the latter two pathways.

GBPs assist caspase-11 activation in response to infections with Gram-negative bacteria (10–12). It was proposed that GBPs lyse vacuoles containing Gram-negative bacteria and thereby release LPS into the host cell cytosol (10). However, we observed that GBPs were able to promote caspase-11 activation in response to *Chlamydia muridarum* infections without any detectable recruitment of GBPs to *Chlamydia*-containing vacuoles, thus arguing against the vacuolar lysis model (11). In addition, we observed that GBPs accelerated caspase-11 activation in cells transfected with LPS aggregates (12), demonstrating that GBPs can impact the kinetics of caspase-11 activation independently of an infection. Because of the recent discovery that Gram-negative OMVs serve as vehicles for the delivery of LPS into the host cell cytosol (15), we hypothesized and here demonstrate a central role for GBPs in the activation of caspase-11 by OMVs.

Bacteria constitutively produce OMVs. Bacterial OMV production is further increased by physiological stressors such as changes in the redox state, nutrient availability, or pH that bacteria experience during cell entry, and especially phagocytosis by macrophages (16, 32–34). We therefore propose that caspase-11 activation during the infection of macrophages with pathogens such as *Salmonella*, *Legionella*, or *Chlamydia* is induced at least in part by the bacterial secretion of OMVs. OMVs released during infection are then processed in a GBP-dependent manner to trigger caspase-11-dependent pyroptosis and canonical inflammasome activation. More detailed studies are required to delineate the mechanism(s) by which GBPs enable caspase-11 activation following OMV treatment. Considering that GBPs are part of the membrane-remodeling dynamin protein superfamily (35), GBPs could potentially play a role in controlling membrane dynamics at the OMV-endosomal interface and thereby expose the lipid A moiety of LPS toward the cytosolic face of endosomes. Extensive cell biological and biochemical studies will be required to test this and alternative hypotheses regarding the molecular mechanism by which GBPs promote caspase-11 activation in OMV-exposed macrophages.

Whereas the addition of purified LPS to culture medium is insufficient to trigger robust caspase-11 activation in wild-type BMDMs, injection of LPS into mice induces caspase-11-dependent sepsis (4, 5). Therefore, circulating LPS *in vivo* is most likely ingested and presented to caspase-11 by designated cell types. Hepatic macrophages (Kupffer cells), sinusoidal endothelial cells, and hepatocytes are credited to be mainly responsible for the removal and detoxification of LPS from the bloodstream (36–38) and therefore are also candidates to mediate *in vivo* caspase-11 activation in response to circulating LPS. Our study demonstrates that GBPs play a critical role in the host response to circulating LPS. Future studies will need to define how the host promotes caspase-11 activation in response to free LPS *in vivo* and the specific roles that GBPs play in this process.

GBPs were shown to act as positive regulators of infection-induced caspase-11 activation (10, 12), and yet their precise functional role in this process has remained poorly defined (7–9). Our study delineates a unique role for GBPs in controlling caspase-11 activation in response to the sterile delivery of OMVs or free LPS *in vivo*. Our report therefore sets a novel framework within which the cellular and molecular activities of GBPs as regulators of inflammasome activation can be explored.

## MATERIALS AND METHODS

### Mice and cell culture.

Wild-type C57/BL6J mice were originally purchased from Jackson Laboratories and maintained at animal facilities at Duke University Medical Center. *GBP*^chr3−/−^, *GBP5*^−/−^, and *Casp11*^−/−^ mice were previously described (19, 23, 39). *GBP2*^−/−^ mice were generated by the Helmholtz Zentrum München using C57/BL6-derived JM8.N4 mouse embryonic stem cells. Animal protocols were approved by the Institutional Animal Care and Use Committees at Duke University. We used approximately equal numbers of male and female mice for all experiments. Although analyses were performed in the whole group, males and females were also analyzed as subgroups and, based on these analyses, phenotypes were sex independent. BMDMs were derived from mouse femur bone marrow. BMDMs were cultured in tissue culture-nontreated plates in RPMI 1640 with 2-mercaptoethanol, 20% heat-inactivated fetal bovine serum (FBS), and 14% conditioned medium containing macrophage colony-stimulating factor. Three days later, 10 ml of additional medium was added to cells. Cells were cultured for an additional 3 to 4 days before use in experiments.

### Bacterial culture, infection procedures, OMV isolation, and treatments.

*E. coli* K-12 BW25113, the background strain of the Keio collection, was used for all experiments (40). *E. coli* was grown in Luria-Bertani broth (LB) overnight at 37°C. For infection of BMDMs, *E. coli* was diluted in Opti-MEM (Gibco) and added to cells. Cells were centrifuged at 700 × *g* for 10 min. Gentamicin was added to cells 1 h postinfection to a final concentration of 100 µg/ml. For isolation of OMVs, LB was inoculated (1:250 dilution) from bacterial cultures grown overnight at 37°C and cells were grown overnight once more at 37°C (∼18 h). Cells were pelleted with a Beckman Avanti J-25 centrifuge (JLA-10.500 rotor, 10,000 × *g*, 10 min, 20°C), and the supernatants were filtered through a 0.45-µm low-protein-binding Durapore membrane (polyvinylidene fluoride; Millipore). Filtered supernatants were either centrifuged again with the Beckman Avanti J-25 centrifuge (JLA-16.250 rotor, 38,400 × *g*, 3 h, 20°C) to pellet OMVs or, for higher culture volumes, concentrated through a 100,000-kDa filter with a tangential-flow filtration system (Millipore) to a final volume of 250 ml and filter sterilized again. In both cases, another centrifugation step followed using the Beckman Optima TLX Ultracentrifuge (TLA 100.3 rotor, 41,000 × *g*, 1 h, 20°C). The supernatant was aspirated, and OMV pellets were resuspended in phosphate-buffered saline (PBS) and subsequently filter sterilized through 0.45-µm Ultra-free spin filters (Millipore). OMV preparations were monitored for sterility by streaking a portion of the sample on LB agar and incubated at 37°C overnight. Protein concentration was measured via Bradford protein assay. For treatment of BMDMs, OMVs or LPS (Ultra-pure LPS from *Salmonella enterica* serovar Minnesota R595; List Biological Laboratories) was diluted in Opti-MEM (Gibco) and added directly to cells. *Legionella pneumophila* experiments were conducted by using a flagellin-deficient LP01 strain (41). Bacteria were grown in broth culture to an optical density at 600 nm (OD_600_) of 3.5 to 4. For infection of BMDMs, *L. pneumophila* was diluted in Opti-MEM and added to cells. Cells were then centrifuged at 700 × *g* for 10 min. Medium was removed and replaced with fresh Opti-MEM 1 h postinfection.

### Colony-forming assay.

IFN-γ-primed (100 U/ml) BMDMs were infected with *E. coli* at an MOI of 25. At 1 h and 4 h postinfection, bacteria were harvested following lysis of cells with PBS plus 0.01% Triton X-100. Serial dilutions of bacteria were plated on LB agar plates, and total CFU were determined.

### Cytotoxicity assays and *in vitro* cytokine measurements.

Naive or IFN-γ-primed (100 U/ml) BMDMs were treated with 2 µg/ml OMVs or infected with *E. coli* at an MOI of 25 unless concentrations were otherwise indicated. To measure cytotoxicity, 8 h after treatment with OMVs or *E. coli* infection lactate dehydrogenase (LDH) release was measured using the CytoTox One homogenous membrane integrity assay (Promega), essentially as described previously (12). Relative LDH release was calculated with the following formula: (sample − untreated control)/(lysed control − untreated control) × 100. Alternatively, cytotoxicity was measured as a function of propidium iodide (PI) uptake. Experiments were carried out in Opti-MEM containing 6 µg/ml PI. Measurements were read at indicated time points on an Enspire 2300 (PerkinElmer) multilabel reader, as described previously (12). For determining cytokine concentrations, supernatants were collected 8 h posttreatment or postinfection. IL-1β and IL-18 levels were measured via enzyme-linked immunosorbent assay (ELISA; eBioscience), as described previously (11).

### Immunocytochemistry and analysis.

For visualizing ASC puncta, IFN-γ-primed BMDMs were infected with *E. coli* at an MOI of 25 or treated with 2 µg/ml OMVs for 8 h. Following infection/treatment, cells were fixed for 5 min with ice-cold methanol. Cells were blocked with 5% bovine serum albumin (BSA) (Amresco) for 30 min and then incubated with rabbit anti-ASC antibody (AG-25B-0006; Adipogen) overnight at 4°C. Cells were then washed three times with PBS-saponin (0.1%)-BSA and stained with anti-rabbit Alexa Fluor 568-conjugated secondary antibody (Invitrogen) and Hoechst stain for 1 h at room temperature (RT). Per replicate, ASC puncta were quantitated for >300 cells. For GBP2-LPS colocalization experiments, IFN-γ-primed BMDMs were treated with 2 µg/ml OMVs for 2 h. Cells were fixed in 4% (wt/vol) paraformaldehyde for 15 min at RT. Cells were washed 3 times with PBS and permeabilized with 0.1% Triton X-100 for 10 min, before blocking with 2.5% BSA in PBS for 30 min at RT. Cells were incubated with anti-GBP2 (42) and anti-LPS antibodies (DS-MB-01267; RayBiotech) for 1 h at RT. Following incubation with primary antibodies, cells were stained with Alexa Fluor-conjugated secondary antibodies and Hoechst stain for 1 h at RT. Line trace analysis was performed with Fiji software (ImageJ; National Institutes of Health), as described previously (42). Imaging was performed using a Zeiss Axioskop 2 upright epifluorescence microscope or a Zeiss 780 upright confocal microscope.

### Immunoblotting.

Total protein from lysates and supernatants was analyzed via immunoblotting. IFN-γ-primed BMDMs were challenged with *E. coli* infection or OMVs for 8 h, supernatants were collected, and protein was concentrated via trichloroacetic acid (TCA) precipitation. A 100% (wt/vol) TCA solution in water was added to supernatants to a final TCA concentration of 10%. Samples were incubated on ice overnight. Following incubation, samples were centrifuged at 21,130 × *g* for 10 min. Supernatant was removed, and pellets were rinsed with acetone twice. Pellets were then air dried and suspended in 8 M urea. Whole-cell extracts were prepared by lysing cells in RIPA lysis buffer (Sigma-Aldrich). Samples were loaded on a 4 to 20% gradient SDS-PAGE gel and transferred to polyvinylidene difluoride (PVDF). Membranes were blocked in Tris-buffered saline–0.1% Tween 20 (TBST) with 5% BSA or 5% nonfat dry milk. Membranes were incubated with primary antibodies overnight at 4°C, and secondary antibody incubations were performed for 1 h at RT. The following primary antibodies and dilutions were used: rabbit anti-caspase-1 (AG-20B-0042; Adipogen; 1:1,000), goat anti-IL-1β (AF-401-NA; R&D Systems; 1:1,000), and mouse anti-β-actin (A2228; Sigma-Aldrich; 1:1,000).

### RNA isolation and qPCR expression analysis.

Spleen RNA was isolated using TRIzol reagent (Thermo Fisher) according to the manufacturer’s instructions. RNA was further purified using the RNeasy minikit (Qiagen). Reverse transcription was achieved using the iScript cDNA synthesis kit (Bio-Rad Laboratories), and quantitative PCR (qPCR) was performed with PerfeCTa SYBR green FastMix (Quanta Biosciences) using a 7500 Fast real-time PCR system (Applied Biosystems). Relative mRNA levels were calculated following normalization against transcript levels of glyceraldehyde-3-phosphate dehydrogenase (GAPDH). The following primers were used: mGBP2 F, 5′-CTGCACTATGTGACGGAGCTA-3′; mGBP2 R, 5′-GAGTCCACACAAAGGTTGGAAA-3′; mGAPDH F, 5′-GGTCCTCAGTGTAGCCCAAG-3′; mGAPDH R, 5′-AATGTGTCCGTCGTGGATCT-3′.

### *In vivo* challenges.

All mice used for *in vivo* challenges were at the age of 8 to 12 weeks. For OMV challenge experiments, mice were injected intraperitoneally (i.p.) with poly(I⋅C) at a dose of 2 mg/kg body weight or PBS control and then 6 h later injected i.p. with 4 µg purified OMVs per mouse or an equal volume of PBS control. Serum was obtained 6 h after OMV injection. For LPS challenge experiments, mice were injected i.p. with LPS (*E. coli* O111:B4 LPS; L3024; Sigma) at a dose of 8 mg/kg body weight or PBS control. Serum was obtained 4 h postinjection. Serum IL-1β and IL-18 concentrations were measured by ELISA. For the study of lethal endotoxemia, mice were first challenged i.p. with poly(I⋅C) (2 mg/kg) followed 6 h later by i.p. injection of LPS (20 mg/kg). Mice were monitored every 3 h for 48 h following initial injection. Mice were considered moribund and euthanized if they dropped below 80% starting weight or if they exhibited severe ataxia, indicated by lack of righting response.

### Statistical analyses.

Data analysis was performed using GraphPad Prism 6.0 software. Data shown are means ± standard errors of the means (SEM) unless otherwise indicated. Statistical significance was calculated using the two-way analysis of variance (ANOVA) (with Tukey’s or Sidak’s multiple-comparison test), unless otherwise noted in figure legends.
