# Supplementary figures and images for: Inflammasome Activation by Bacterial Outer Membrane Vesicles Requires Guanylate Binding Proteins
Source: mBio. 2017 Oct 3;8(5):e01188-17. doi: 10.1128/mBio.01188-17 (PMC5626967; doi:10.1128/mBio.01188-17)

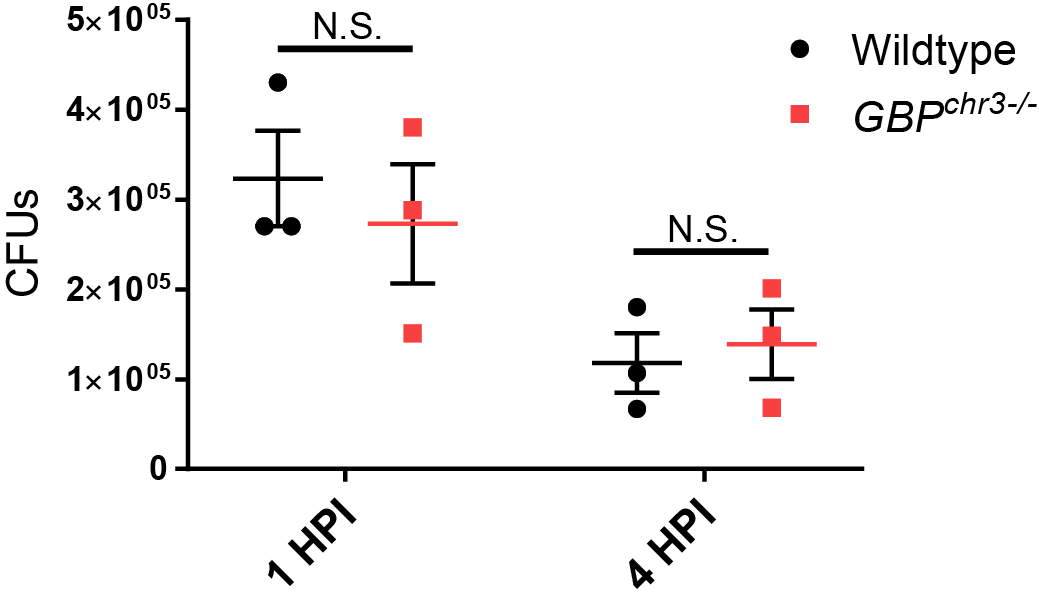

Supplement: FIG S1 [file mbo005173522sf1.tif]

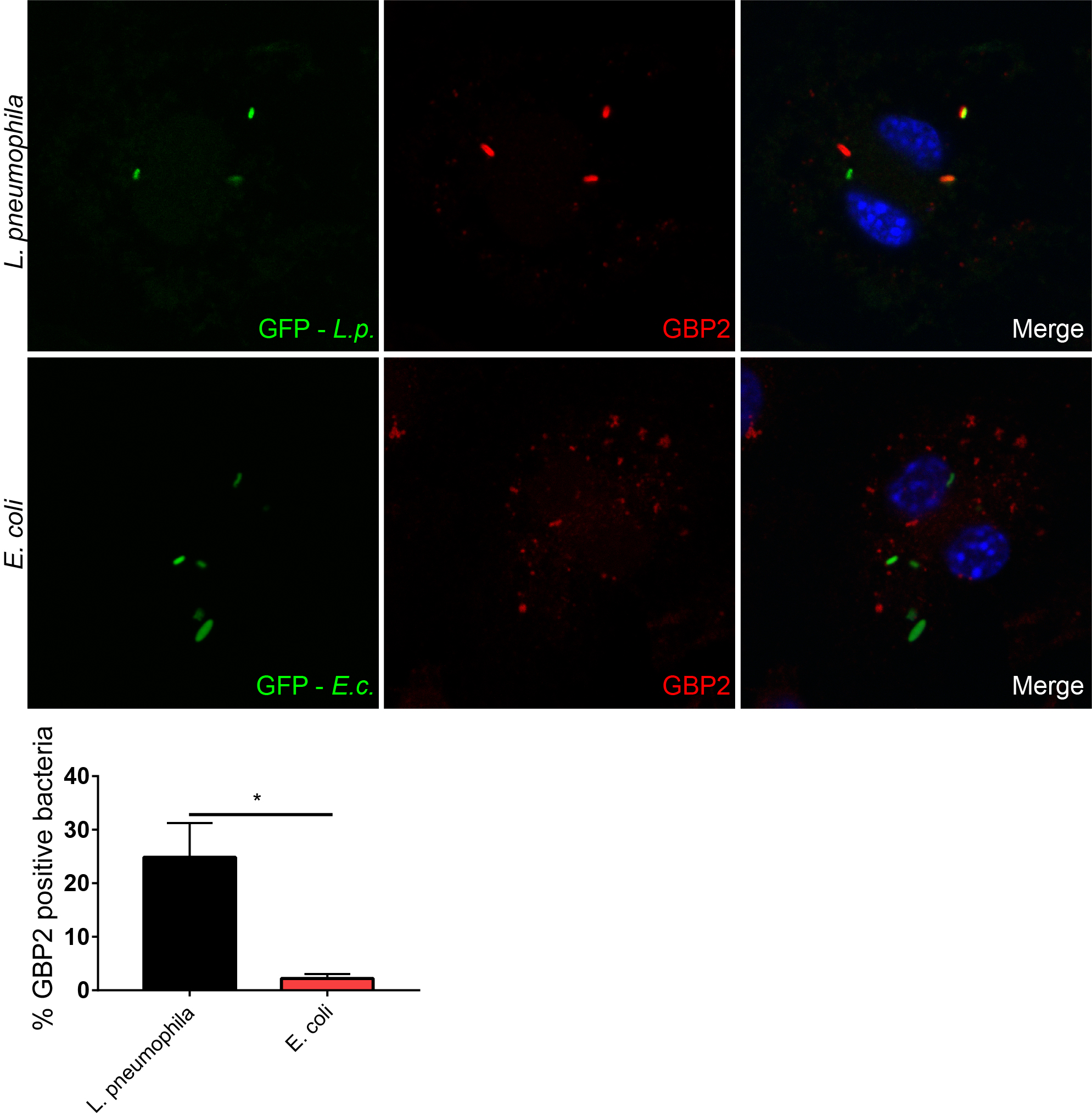

Supplement: FIG S2 [file mbo005173522sf2.jpg]

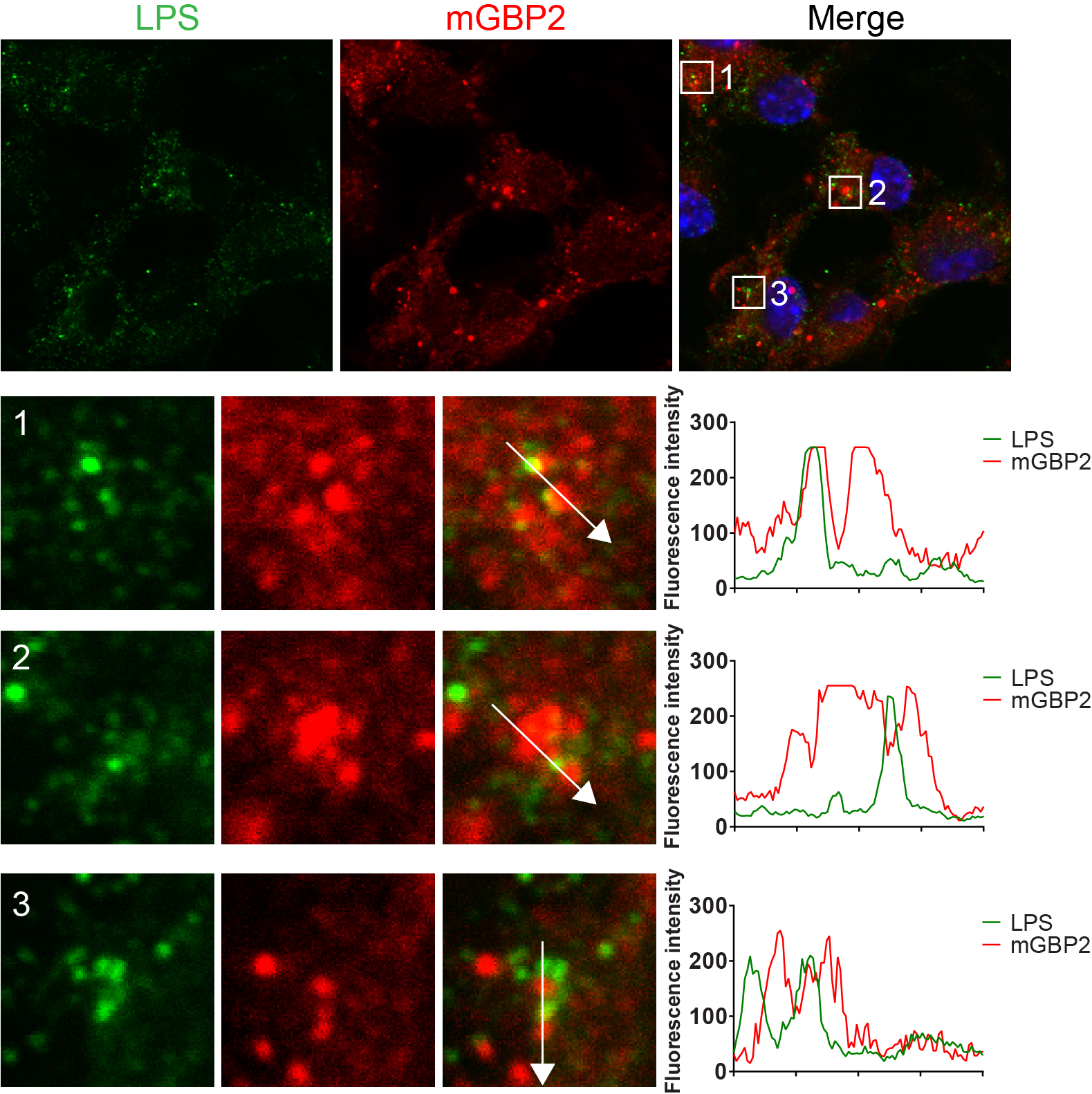

Supplement: FIG S3 [file mbo005173522sf3.jpg]

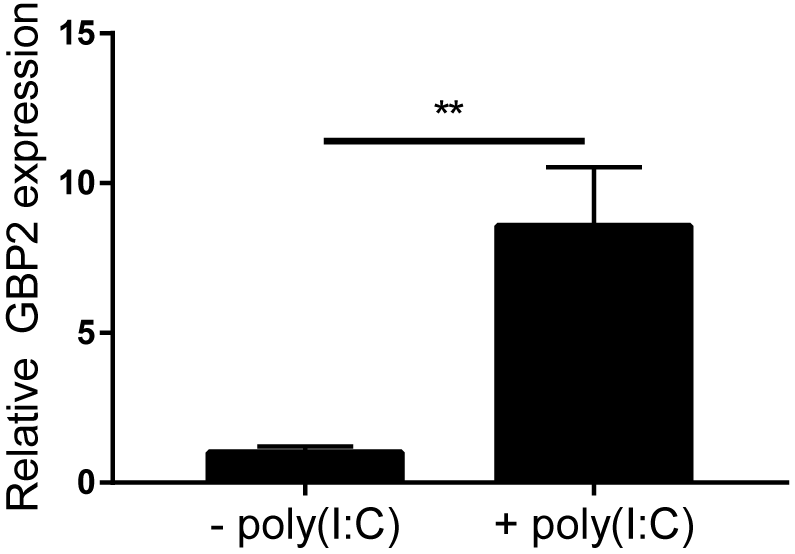

Supplement: FIG S4 [file mbo005173522sf4.tif]

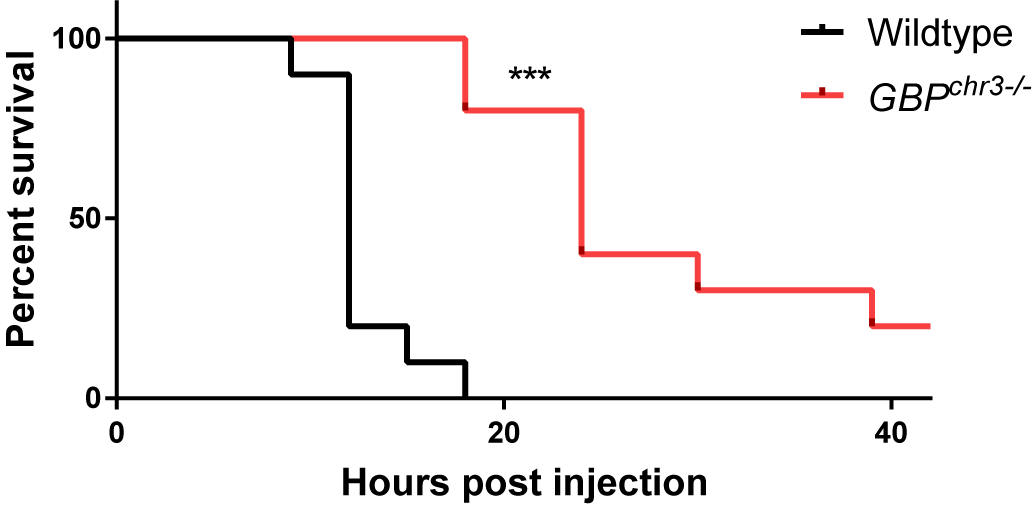

Supplement: FIG S5 [file mbo005173522sf5.tif]
